# Fe(2)OG: an integrated HMM profile-based web server to predict and analyze putative non-haem iron(II)- and 2-oxoglutarate-dependent dioxygenase function in protein sequences

**DOI:** 10.1186/s13104-021-05477-z

**Published:** 2021-03-01

**Authors:** Siddhartha Kundu

**Affiliations:** Department of Biochemistry, All India Institute of Medical Sciences, Ansari Nagar, New Delhi, 110029 India

**Keywords:** Facial triad, Hidden markov model, Non-haem iron(II)- and 2-oxoglutarate-dependent dioxygenases

## Abstract

**Objective:**

Non-haem iron(II)- and 2-oxoglutarate-dependent dioxygenases (i2OGdd), are a taxonomically and functionally diverse group of enzymes. The active site comprises ferrous iron in a hexa-coordinated distorted octahedron with the apoenzyme, 2-oxoglutarate and a displaceable water molecule. Current information on novel i2OGdd members is sparse and relies on computationally-derived annotation schema. The dissimilar amino acid composition and variable active site geometry thereof, results in differing reaction chemistries amongst i2OGdd members. An additional need of researchers is a curated list of sequences with putative i2OGdd function which can be probed further for empirical data.

**Results:**

This work reports the implementation of $Fe\left(2\right)OG$, a web server with dual functionality and an extension of previous work on i2OGdd enzymes $\left(Fe\left(2\right)OG\equiv \{H2OGpred,DB2OG\}\right)$. $Fe\left(2\right)OG$, in this form is completely revised, updated (URL, scripts, repository) and will strengthen the knowledge base of investigators on i2OGdd biochemistry and function. $Fe\left(2\right)OG$, utilizes the superior predictive propensity of HMM-profiles of laboratory validated i2OGdd members to predict probable active site geometries in user-defined protein sequences. $Fe\left(2\right)OG$, also provides researchers with a pre-compiled list of analyzed and searchable i2OGdd-like sequences, many of which may be clinically relevant. $Fe(2)OG$, is freely available (http://204.152.217.16/Fe2OG.html) and supersedes all previous versions, i.e., H2OGpred, DB2OG.

## Introduction

**Fig. 1:**
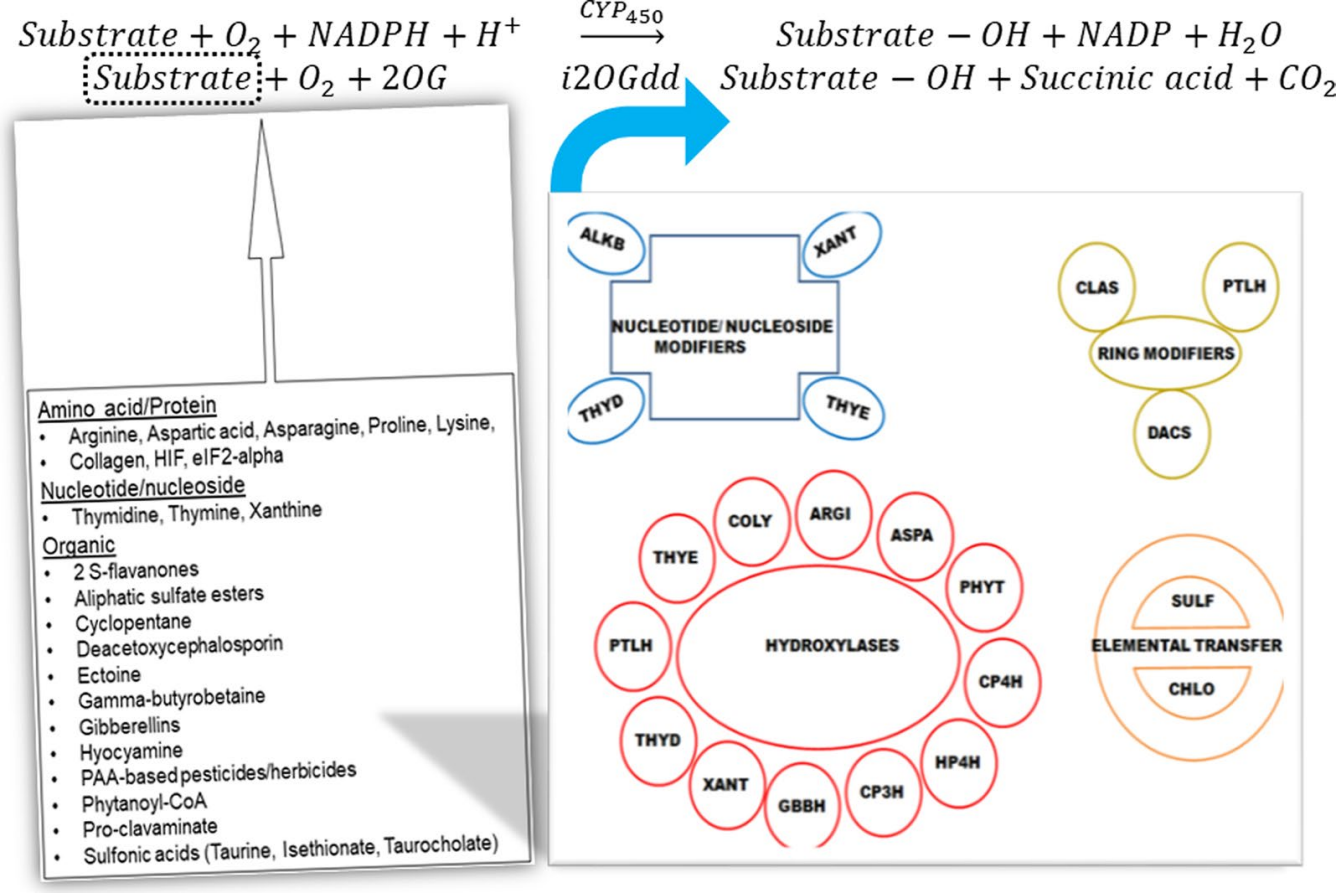
Non-haem iron(II)- and 2-oxoglutarate-dependent dioxygenase based catalysis. This large superfamily of enzymes is characterized by a variable reaction chemistry, broad spectrum of substrates and is present in all kingdoms of life. The enzymes are characterized by a triad of Histidine, Aspartic-/Glutamic-acid and Histidine residues which co-ordinate ferrous iron. 2OG and the substrate. Enzymes are classified on the basis of the substrate(s) transformed and dominant reaction chemistry. Each cluster is a HMM-profile of at least two members and is derived from enzymes with available empirical data (structure, kinetic, mRNA expression). *ALKB* Alk-B like demethylase, *ARGI* Arginine hydroxylase; *ASPA* Aspartyl:Asparaginyl hydroxylase, *CHLO* Chlorinating enzyme, *CLAS* Clavaminate synthase; *COLY* Collagen lysyl dioxygenase, *CP3H* Collagen prolyl 3-hydroxylase, *CP4H* Collagen prolyl 4-hydroxylase, *CYCL* Cyclization and ring closure, *DACS* Deacetoxycephalosporin-C synthase, *DSAT* Desaturases, *ECTO* Ectoine hydroxylase, *FLAV* 2S-Flavones, *GBBH* γ-butyrobetaine hydroxylase; *GIAC* Gibberellic acid modification, *HILY* Histone lysyl demethylase, *HP4H* Hypoxia prolyl 4-hydroxylase, *HYOS* Hyoscyamine hydroxylase, *NUHY* Nucleotide/nucleoside hydroxylase, *OGFD* Eukaryotic initiation factor 2α, *PHYT* Phytanoyl-CoA hydroxylase, *PTLH* 1-Deoxypentalenic acid 11β-hydroxylase, *SULF* Sulfate cleaving, *TFDA* 2,4-Diphenoxyacetic acid metabolizing, *THYD* Thymdine dioxygenase, *THYE* Thymine dioxygenase, *XANT* Xanthine hydroxylase

Dioxygenases, unlike monooxygenases are oxidoreductases which can incorporate both atoms of molecular oxygen, one each into a substrate and co-substrate (Fig. 1). These enzymes are classified on the basis of a metal co-factor (iron, cobalt, nickel, copper) and the presence of a haem-prosthetic group. Iron-based dioxygenases are mononuclear (extradiol catechol, $EC \mathrm{1.13.11},x$; 2-oxoglutarate-dependent, $EC \mathrm{1.14.11},x$), possess Rieske clusters (naphthalene 1,2-dioxygenase, $EC \mathrm{1.14.12,12}$) and may utilize haem (indoleamine 2,3-dioxygenase, $EC \mathrm{1.13.11,52}$; tryptophan 2,3-dioxygenase, $EC \mathrm{1.13.11,11}$) [1–5]. Extradiol and 2OG-dependent dioxygenases, possess a triad of catalytically competent $H{X}_{n}[DE]{X}_{n}H$ residues and comprise one face of a distorted octahedral co-ordination sphere with iron(II) [3–6]. The other face is formed by three displaceable water molecules, a factor that contributes significantly to the architecture of the active site [3–6]. The subset that comprises non-haem iron(II)- and 2OG-dependent dioxygenases (i2OGdd) is characterized by $HX\left[DE\right]{X}_{n}H \left(n\in [\mathrm{50,120}]\right)$, in a jelly-roll or double stranded beta-helical fold [4–6]. The i2OGdd-superfamily $(EC \mathrm{1.14.11}.x)$, is characterized by the dependence of catalysis on 2-oxoglutarate and occurs by the bi-dentate co-ordination of $C1$ (carboxylic acid) and $C2$ (2-oxo/alpha-keto) with mononuclear ferrous iron (Fig. 1). The last dative bond is the cognate substrate after the solitary water molecule has been displaced. The reaction chemistry involves a progressive increase in the oxidation state of iron ($II\to IV$) and is followed by proton abstraction and the formation of a substrate-radical. This in turn leads to catalytic conversion of the substrate by the incorporation of a single oxygen atom (Fig. 1). The transformation is therefore, a de facto oxidative hydroxylation although, this is accompanied in most cases by a concomitant desaturation, cyclization, stereo-isomerization and sulfate cleavage (Fig. 1) [7–9]. The second oxygen atom is incorporated into 2OG with the release of succinic acid and ${CO}_{2}$ (Fig. 1). Members of the i2OGdd-superfamily participate in cell signaling under hypoxic conditions, DNA repair, stress response mechanisms, metabolism (lipids, growth factors) and biodegradation of herbicides (Fig. 1) [5, 10–17].

The work presented revises, updates and integrates the functionality of two servers, i.e., $Fe(2)OG\equiv \left\{H2OGpred, DB2OG\right\}$ [18, 19]. $Fe(2)OG$, can be used by researchers as a single-point web resource to screen protein sequence(s) for potential i2OGdd-activity and shortlist putative i2OGdd members from the available pre-compiled sequence repository. The latter is searchable on the basis of taxonomy, cellular compartment and HMM-profiles of the sequences. A novel feature of $Fe\left(2\right)OG$ is the inclusion of clinically relevant non-haem iron(II)- and 2OG-dependent dioxygenases. This includes links and preliminary analyses to several human putative i2OGdd members. The coding and interfacing is done using in-house developed PERL scripts.

## Main text

### Rationale for incorporating empirical data into a profile-based search application

Non-haem iron(II)- and 2OG-dependent-dioxygenases are characterized by variable reaction chemistry and a broad spectrum of substrates. The reverse mapping of substrate descriptors to the active site of known enzymes is well documented and can be utilized to repurpose pharmacological agents. Several theoretically sound statistical tools such as multi-class support vector machines (SVMs), artificial neural networks (ANNs), and hidden markov models (HMMs) have been utilized to garner insights into the active site geometry of an enzyme in the presence of a pharmacophore [18–22]. Although HMMs, as a predictive modality are non-committal, this can be rectified by mathematical filters. The transformed output can then be utilized by clustering algorithms and ANNs to generate unambiguous predictors [21, 23, 24]. In fact, a rigorously derived integrated HMM-ANN algorithm has been presented and used to characterize sequences which are few and closely related such as those from an enzyme family or sub family [23, 24].

### Mathematical basis for the algorithms deployed by $Fe\left(2\right)OG$

Whilst, a detailed description of the computational pipeline deployed and its relevance has already been published, the mathematical basis for these has not been addressed [18, 19]. Briefly, HMM-profiles of catalytically relevant clusters and laboratory validated enzymes of the i2OGdd-superfamily $\left({a}_{i}\in A\subseteq \mathcal{H}\right)$ are utilized to score regions of an amino acid sequence. The empirical data that is considered is the presence of one or more 3D-structures, kinetic and mutagenesis data and mRNA expression levels [18]. A suitable mathematical representation is as under:

1 $$\begin{gathered} A = \{ a_{1} ,a_{2} , \ldots a_{i} |\# a_{i} \ge 2\} \hfill \\ = \bigcup {a_{i} } \hfill \\ \end{gathered}$$

#### Theorem:

A unique set of HMM profiles $\left(A,B\subseteq \mathcal{H}\right)$ can exist iff there is at least one unique sub-profile.

$$\begin{array}{cccc}A\ne B& iff& \#({a}_{i}\cap {b}_{i})\le min\left(\#{a}_{i},\#{b}_{i}\right)& {a}_{i}\in A\subseteq \mathcal{H},{b}_{i}\in B\subseteq \mathcal{H},\left\{{\#a}_{i},{\#b}_{i}\right\}\ge 2\end{array}$$

#### Proof:

$$\begin{array}{*{20}c} {Case1} & {if \cap _{{i = 1}} a_{i} = \emptyset , \cap _{{i = 1}} b_{i} = \emptyset } & {} \\ {SinceA,B \subseteq {\mathcal{H}},} & {A \cap B \ne \emptyset } & {} \\ {Rewriting,} & {\left( { \cup _{{i = 1}} a_{i} } \right) \cap \left( { \cup _{{i = 1}} b_{i} } \right) \ne \emptyset } & {} \\ {Expanding,} & {\left( {a_{1} \cap b_{1} } \right) \cup \left( {a_{2} \cap b_{2} } \right) \ldots \left( {a_{i} \cap b_{i} } \right) \ne \emptyset } & {} \\ {Let,} & {\left( {a_{1} \cap b_{1} } \right) \cup \left( {a_{2} \cap b_{2} } \right) \ldots \left( {a_{i} \cap b_{i} } \right) = k} & {} \\ {} & {k \in \left( {a_{1} \cap b_{1} } \right)ORk \in \left( {a_{2} \cap b_{2} } \right) \ldots ORk \in \left( {a_{i} \cap b_{i} } \right)} & {} \\ {Clearly,} & {k = a_{i} \cup b_{i} } & {} \\ {Generalizing,} & {\exists \left( {a_{i} \cap b_{i} } \right)|\# (a_{i} \cap b_{i} ) \le min(\# a_{i} ,\# b_{i} )} & {(a)} \\ {Case2} & {if \cap _{{i = 1}} a_{i} \ne \emptyset , \cap _{{i = 1}} b_{i} \ne \emptyset } & {} \\ {Let,} & { \cap _{{i = 1}} a_{i} = a_{i} \in A|a_{i} \ge 2, \cap _{{i = 1}} b_{i} = b_{i} \in B|b_{i} \ge 2} & {} \\ {itfollows,} & {\# (a_{i} \cap b_{i} ) \le min(\# a_{i} ,\# b_{i} )} & {(b)} \\ {} & {} & {} \\ {From\left( a \right),(b)} & {A \ne B} & {} \\ {} & {} & {} \\ {Conversely,} & {ifA \ne B,} & {} \\ {SinceA,B \subseteq {\mathcal{H}},} & {A \cap B \ne \emptyset } & {} \\ {Let,} & {A \cap B = a_{i} \cup b_{i} } & {} \\ \Rightarrow & {\exists \left( {a_{i} \cap b_{i} } \right) \in A \cap B|\# (a_{i} \cap b_{i} ) \le min(\# a_{i} ,\# b_{i} )} & {} \\ {} & {a_{i} \ne b_{i} } & {} \\ \end{array}$$

$Fe\left(2\right)OG,$ then, is an implementation of a particular instance of the combined HMM of sequences and available structures $\left(A={a}_{i}|1\le i\le 28, 2\le \#{a}_{i}\le 4\right)$ [19]; URL-http://janelia.org. The lower limit of number of the sequences in each profile $\left(\mathrm{min}\left(\#{a}_{i}\right)\right)$ Eq. (1) is implied by definition*.* The upper limit, however, is estimated as a proportion of the total number of sequences,

2 $$\max \left( {\# a_{i} } \right) = \frac{{\# {\mathcal{H}}}}{\# A}$$

### Description and utilization of $Fe(2)OG$

#### i) $Fe(2)OG$, a predictor of the catalytic spectrum of an unknown or single function enzyme

**Fig. 2:**
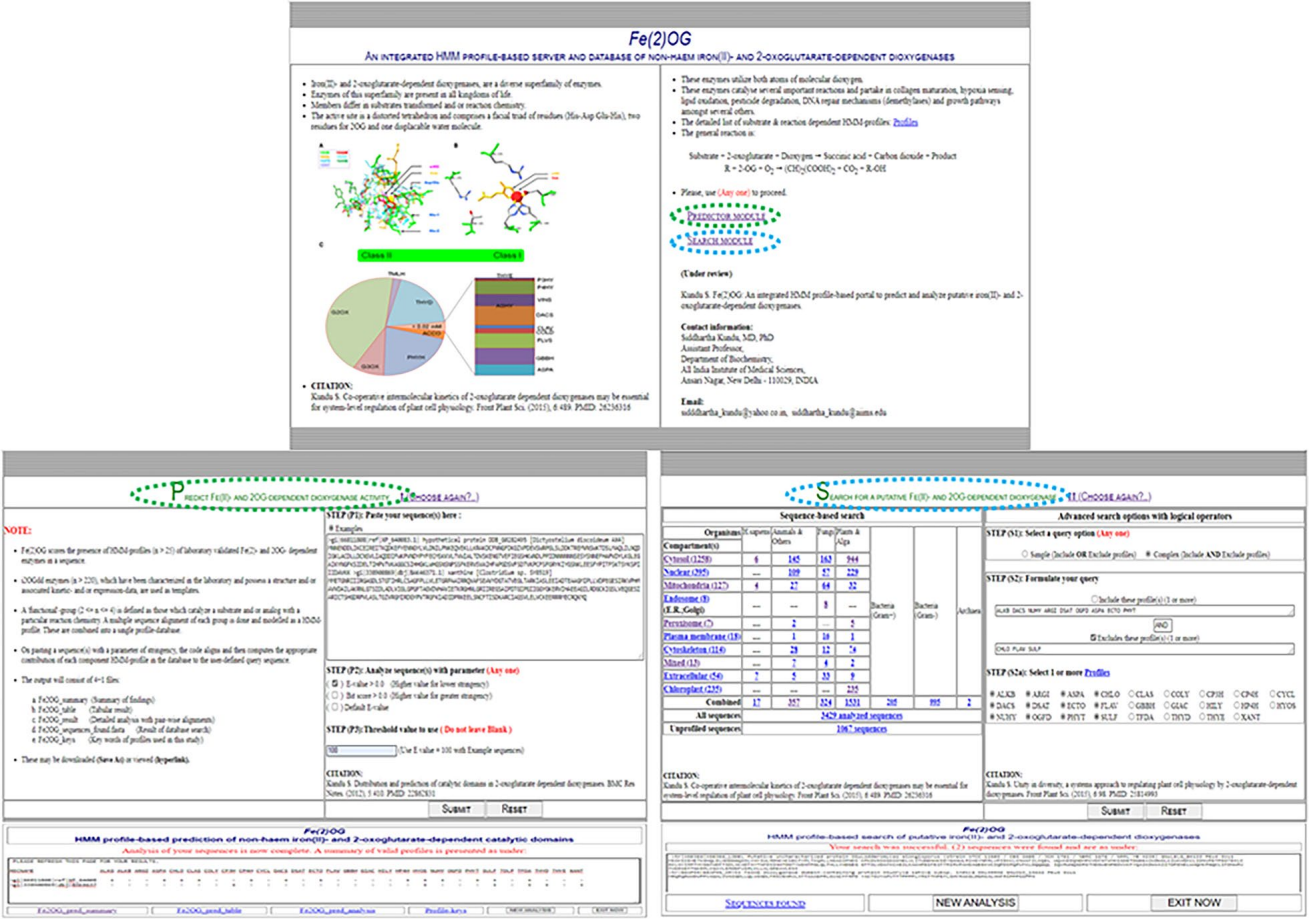
$$Fe\left(2\right)OG$$, an integrated HMM-profile based web server of non-haem iron(II)- and 2OG-dependent dioxygenases. $$Fe\left(2\right)OG$$, is a revised and updated web resource which completely integrates the functionality of two independent servers, $$H2OGpred$$ and $$DB2OG$$. A brief introduction with regards to active site geometry and reaction chemistry is provided. Clear directives on the usage, precautions and processing of data are provided. The predictor and search modules are distinct from each other and can be submitted independently of each other. The predictor module of $$Fe(2)OG$$ is modified and will analyze a user-defined sequence(s) for the complete set of HMM-profiles. Users may use the “Examples” option to add sample sequences or “Paste” their own selection in the text area. These, when submitted will result in a chart of closely-matched HMM-profiles across the full length of the sequence. Since, the output is based on optimally chosen parameters ($$Evalue, Bit score)$$, these need to be manually added and carefully chosen before submission. The database, too, is updated and has been extensively curated. Here, putative i2OGdd members are classified and arranged on the basis of their taxonomy and dominant cellular compartment. These are arranged in an easy-to-select matrix format. The sequences of this database can also be queried and recursively analyzed using the logical operators (AND, OR) for the presence of combinations of HMM-profiles. The results of these searches can be downloaded and investigated further. The list of sequences not analyzed is also presented along with links to several human putative i2OGdd members. Abbreviations: 2OG, 2-oxoglutarate; HMM, Hidden Markov Models; i2OGdd, non-haem iron(II)- and 2OG-dependent dioxygenases; $$H2OGpred$$ HMM-based prediction of putative 2-oxoglutarate function; $$DB2OG$$ database of sequences that results from a generic-2OG HMM-based query of UniprotKB

The algorithm and code that $Fe\left(2\right)OG$ utilizes to predict the dominant profile, in a user-defined sequence(s), has been described in detail [18]. Briefly, i2OGdd enzymes $(n>220)$ with available empirical data (structure, kinetic, mRNA expression) are clustered on the basis of the substrates catalyzed and/or the reaction chemistry (Fig. 1) [18]. The enzymes present in each ‘functional’-group, $\left(2\le \#{a}_{i}\le 4\right)$ Eqs. (1) and (2) are then aligned and assigned a HMM-profile (Figs. 1 and 2) [18]. A database of these HMM-profiles is used to probe the catalytic spectrum of a user-defined sequence as per the stringency specified. Unlike $H2OGpred$, $Fe\left(2\right)OG,$ compares a query sequence(s) with all, rather than isolated HMM-profiles (Fig. 2) [18]. The rationale for this alteration is that since the catalytic profile of an unknown sequence(s) is debatable, a generic analysis rather than a specific one is a better indicator of i2OGdd-like activity. Furthermore, sequences with known function can also be investigated for other reaction chemistries. Clearly, in both cases the analysis with individual profiles is superfluous and may be omitted (Table 1A). The tabulated list of relevant cognate substrates, for each profile is also available and may be used as a reference (Figs. 1 and 2). In addition, to the overt directives of use, users can also sample the functionality of $Fe\left(2\right)OG$ by clicking the “Examples” button $(Step P1)$ (Fig. 2). This loads bonafide i2OGdd sequences into the text area which can be analyzed in accordance with the steps that are outlined subsequently. These include choice of threshold parameter $(Evalue,Bit score)$ and assignment of a suitable numerical value $(Steps P2,P3)$ (Fig. 2). The output comprises a tabular summary of suitably matched profiles with detailed statistics and exhaustive pair-wise alignments of all supra-threshold matches (Fig. 2). Since, $Fe\left(2\right)OG$ has dual functionality, the user can submit this independently $\left(Steps P1-P3\right.\to Submit)$ (Fig. 2).

**Table 1:** Comparative analysis and biomedical relevance of non-haem iron(II)- and 2OG-dependent dioxygenases

A		$Fe\left(2\right)OG$	$H2OGpred,DB2OG$
	Nature and composition Mode of prediction and impact Putative i2OGdd sequences Display and accessibility “Unclassified” sequences “All sequences” search criteria Clinically relevant members	Integrated webserver Generic, improved and extendible Recent and relevant Matrix format (compartments, taxonomy) Yes; Amenable to further investigation Omitted; extended profiles of sequences with known function Yes	Independent servers Specific; restricted NA Simple tabular format No NA No

B	Enzymes ($EC \mathrm{1.14.11}.x)$	Physiological role; Disease biology		Profile(s); References
1	Phytanoyl-CoA hydroxylase $\left(x=18\right)$	Phytanic acid hydroxylation;	Refsum’s disease	PHYT; [18, 27]
2	(HIF) Prolyl hydroxylases $\left(x=\mathrm{2,29}\right)$	Hypoxia-induced Proline hydroxylations	Tumor suppression, ubiquitin-mediated proteasomal degradation of HIF via the Von Hippel-Lindau complex	HP3H, HP4H; [13, 14, 18, 28]
3	(HIF) Asparaginyl hydroxylases $\left(x=\mathrm{16,30}\right)$	Hypoxia-induced Aspartic acid/Asparagine hydroxylations	Traboulsi’s syndrome	ASPA; [18, 29, 30]
4	Collagen Prolyl hydroxylases $\left(x=\mathrm{2,28}\right)$	Assembly of mature collagen	Connective tissue disorders, promoter of metastasis	CP3H, CP4H, OGFD; [18, 31, 32]
5	Procollagen-lysine 5-dioxygenase $\left(x=4\right)$		Kyphoscoliotic Ehlers–Danlos syndrome Bruck syndrome	COLY; [18, 33, 34]
6	JMJD6 (Jumonji-domain containing protein) $\left(x=\mathrm{66,67}\right)$ Histone H3 trimethyl L lysine (4|9) demethylase Histone H3 dimethyl L lysine 36 demethylase $\left(x=27\right)$	Arginine demethylation, Lysine hydroxylation Lysine demethylation	Promoters of metastasis, Developmental disorders,	HILY; [18, 35, 36]
7	DNA demethylases ALKBH-1, 2, 3, 4, 5, 8 $\left(x=\mathrm{33,51,53,54}\right)$	DNA/mRNA-repair after alkylation	Heightened propensity for malignant transformation, disorders of growth and development Decreased atherosclerosis	ALKB; [18, 37, 38]

#### ii) $Fe\left(2\right)OG$, a repository of i2OGdd-like sequences

The second component of $Fe\left(2\right)OG$ is a flat-file database. This comprises a pre-compiled and updated list of i2OGdd-like sequences $({n}_{AB}=4496)$ (Fig. 2). This is accomplished by constructing a generic-HMM after combining representative $\left(n\sim 80\right)$ i2OGdd enzymes from each ‘functional’-group. This is then used to query UniprotKB for probable matches $\left({n}_{AB}\right)$ [19]. The downloaded sequences are analyzed and assigned a dominant cellular compartment $\left({n}_{A}\right)$ [19]. Sequences, which are not amenable to these preliminary investigations are annotated as such $\left({n}_{B}\right)$. Users can download updated lists of these sequences $\left({n}_{A}=3429,{n}_{B}=1067\right)$ (Fig. 2). This is facilitated by arranging the sequences as a matrix of compartments $(p)$ and taxonomy $(q) \left(AB=\left\{{y}_{pqr}\in \left({ab}_{pq}\right);p=10,q=7,r\in {\mathbb{N}}\right\}\right). \, Fe\left(2\right)OG$, also uses the logical operators ($\left\{AND,OR\right\}$) to formulate an advanced HMM profile-based query to partition the sequences ($Step S1$; Fig. 2) [19]. Another modification introduced in $Fe\left(2\right)OG$ is the omission of the “All sequences”-option ($Step S1$) (Fig. 2). The rationale for this amendment, is that users may require sequences specific to one or more HMM-profiles (Figs. 1 and 2). Since, each profile is based on a specific reaction chemistry, users will also possess, a priori, a definitive list of probable ligands to characterize the kinetics of their search result with (Fig. 1, Table 1A). Furthermore, the entire database $\left({n}_{A}\right)$ is accessible with the “OR” and “Include these profile(s)”, if the user so chooses ($Steps S1,S2$) (Fig. 2). The other fraction could not be further classified and is presented only in terms of their respective taxonomies $\left({n}_{B}=1067\right)$. Here, too, the user can submit this independently $\left(Steps S1,S2\right.\to Submit)$ (Fig. 2).

### Comparative analysis and biomedical relevance of $Fe\left(2\right)OG$

Despite the similarity in algorithms and general usage, $Fe\left(2\right)OG$, offers several new and upgraded features (Table 1). These include links to i2OGdd members which are uncharacterized and clinically relevant, whilst offering researchers a tool to extend the catalytic profiles of known enzymes. Additionally, the list of sequences with putative i2OGdd function is updated and non-redundant. The i2OGdd are amongst the largest group of non-haem dioxygenases and can arguably compete in importance with the more established cytochrome P450 (${CYP}_{450}$) superfamily of haem monooxygenases (Fig. 1). The differential activity of i2OGdd members in response to fluctuating concentrations of oxygen and iron also suggest a system-level function in sensing and thence regulating the uptake, utilization and release of these micronutrients [25, 26]. In fact, clinical data is available for several i2OGdd enzymes. This includes phytanoyl-CoA hydroxylase, hypoxia-inducible Proline hydroxylases, collagen modifiers (Proline- and Lysine-hydroxylases) and DNA/mRNA-demethylases (Table 1B) [27–38]. The analysis by $Fe\left(2\right)OG$ results in a small subset $(\approx 24\%,n=17)$ of enzymes and are grouped into mitochondrial, cytosolic and extracellular fractions (Additional file 1: Text S1a). However, a larger proportion $(\approx 76\%,n=53)$ remains unclassified and merits a deeper investigation (Additional file 1: Text S1b).

## Limitations

$Fe\left(2\right)OG$, is an online web resource that is dedicated to expanding the knowledge base of non-haem iron(II)- and 2OG-dependent-dioxygenase superfamily of enzymes amongst scientists and clinicians. $Fe\left(2\right)OG$, can predict whether an unknown protein sequence(s) possesses i2OGdd-activity. It also provides preliminary analyses (taxonomy, cellular compartment) and an analytic tool (sequence-based, logical) to shortlist enzyme candidates from a pre-compiled list of sequences. Since, newer sequences are constantly becoming available, $Fe\left(2\right)OG$ will require constant updates to its core of HMM-profiles and the raw sequences that are queried for putative function, thereof, to remain relevant to the biomedical community. However, since this information is dependent on available empirical data, an annual update might suffice. $Fe\left(2\right)OG$, is also not exhaustive and lacks structural-models and simulation data for its members. These short comings will be addressed in future studies.

## Supplementary Information

**Additional file 1: Text S1.** **a** HMM profiles of analyzed human i2OGdd-like sequences (n = 17). **b** Uniprot IDs of unprofiled human i2OGdd-like sequences (n = 53)

## Data Availability

Data is available as supporting material with the manuscript.

## Abbreviations

Fe: Iron
2OG: 2-Oxoglutarate
HMM: Hidden Markov Model
i2OGdd: Non-haem iron(II)- and 2-oxoglutarate-dependent dioxygenases
